# Flower traits and breeding system of *Rhododendron platypodum* diels, an endangered plant species in China

**DOI:** 10.1371/journal.pone.0319513

**Published:** 2025-03-27

**Authors:** Chaoying Wang, Yingzan Xie, Lihui Ma

**Affiliations:** 1 School of Culture and Tourism, Chongqing City Management College, Chongqing, China; 2 Chongqing Urban Ecosystem National Positioning Observation and Research Station, Chongqing Academy of Forestry Sciences, Chongqing, China; Nuclear Science and Technology Research Institute, IRAN, ISLAMIC REPUBLIC OF

## Abstract

*Rhododendron platypodum* Diels is an endangered ornamental plant distributed in the high-elevation subtropical regions of China. Known as one of the “queen flower”, its population is restricted to three sites in China, with only few individuals. To explore the reasons for poor population regeneration and provide theoretical basis for genetic breeding to support its popularization and application of this native garden tree species, field investigation and artificial pollination experiment were conducted to test the flowering characteristics and breeding system of *R. platypodum* in Zhaoyun Mountain, Chongqing. The results revealed that: (1) the flowering period of *R. platypodum* began in mid-April and ended in late May, lasting 36 days, with an average flowering duration of 9.15 days per flower. (2) Pollen viability was highest in the morning and evening, peaking on third day after flowering, while stigma receptivity was highest between the second and third days. (3) The value of hybridization index and the pollen-ovule ratio indicated a partial self-compatibility and facultative outbreeding of this species. (4) Artificial pollination experiments showed no evidence of parthenogenesis or automatic self-pollination, and the fruit set rates for xenogamy, geitonogamy were higher than those for self-pollination, with artificial pollination yielding higher fruit set rate than natural pollination. (5) The primary effective pollinators of *R. platypodum* were identified as *Bombus* sp. and *Apis cerana* sinensis. Our research found that instead of long flowering period, high pollen viability and simultaneous mature of stigma and pollen, high geitonogamy relying on pollinators for effective production and the pollen restriction are more likely to have adverse effects on the population of *R. platypodum*. Further factors such as limitation in seed dispersal, germination challenges, and environmental requirement for seedlings and saplings may contribute to the endangerment of *R. platypodum*.

## Introduction

Reproduction is a critical phrase in the life cycle of plants, serving as a fundamental guarantee for the survival and propagation of plant species. To understand the mechanism underlying the endangerment of a species, studying its reproductive biology is essential [1,2]. The plant breeding system, comprising floral morphological traits, floral organ expression, flower opening degree and style, self-compatibility and mating system, significantly influences plant phenotypes and evolutionary route [3–5]. The breeding process, along with pollinator and pollination modes, plays a decisive role in shaping the genetic composition and fitness of plant offspring [6–9]. Flowering plant reproduction primarily depends on their mating system, which encompass all supporting organs involved in gamete fusion and zygote formation, especially their reproductive organs [10]. Therefore, factors such as the flower type, morphological characteristics, color, blooming mode, blooming time, and traits affect the efficiency and reproduction success [11–13]. In addition to human interference, reproductive disorders are among the leading causes plants endangerment [9,14,15]. For instance, low fruit and seed setting rates are key factors in the endangerment of species like *Emmenopterys henryi* Oliv. [16], *Xanthoceras sorbifolium* Bunge [17], *Styrax zhejiangensis* [18] and *Buchanania lanzan* [19]. These challenges often arise from the loss of reproductive organs function, or limitation in the pollination processes [20].

*Rhododendron* is one of the world’s four major garden flowers and often known as the “king of woody flowers”, comprises more than 900 species distributed in Asia, with 576 species found in China, over 74% of which are endemic [21]. The southwestern Hengduan Mountains serve as the richest distribution and evolutionary center of *Rhododendron* plants. Numerous studies have been conducted on the floral characteristics, flowering dynamics, and breeding systems of various *Rhododendrons* species [22–31]. These studies reveal that the breeding systems of most *Rhododendron* species favor hybrid mating system predominantly involving outcrossing [21], while species such as *Rhododendron sinofalconeri* [32] and *Rhododendron longipedicellatum* [33] exibit mixed mating system, incorporating both self-crossing and outcrossing. However, no research has been reported on the breeding system of *R. platypodum*.

*Rhododendron platypodum* is an evergreen shrub or small tree that grows on rocky terrains or in dense forests at high altitudes. This species is listed as vulnerable (VU) in *China Red List of Biodiversity - Higher Plants Volume* [34] and the *Red List of Rhododendrons* [35]. However, Wang et al. [36] reassessed its conservation status and classified it as endangered (EN) species. Studies on *R. platypodum* have revealed that it exists in only three populations in China, distributed across the steep cliffs and ridges of Jinfo Mountain, Zhaoyun Mountain and Baima Mountain in Chongqing, with a total population of approximately 377 individuals [37]. Typical community associations include *R. platypodum* +  *Fargesia spathacea* and *R. platypodum* +  *Litsea cubeba* [38]. Although the diameter class of the population is complete, some survey quadrats lacked small seedlings [37,38]. The population characteristics of *R. platypodum* are significantly influenced by many environmental factors, especially the surface transmittance and soil type on seedling density and the surface transmittance on plant flowering [37]. These indicated that the poor habitat may have some influence on its reproductive ecology.

Research on pollination ecology is crucial for understanding the potential regeneration bottleneck of endangered species [1], as pollen and seed dispersal, which underpin population regeneration and structure maintenance [39,40], are highly affected by environment conditions. This process is often considered to be “the most vulnerable stage in the plant life cycle” [41]. Consequently, studying the reproductive ecology of such species is essential for ensuring continuous population regeneration [42]. This study investigated the flowering characteristics and breeding systems of *R. platypodum* through field investigation and artificial pollination experiment conducted in Zhaoyun Mountain, Chongqing, to provide a theoretical basis for its conservation promotion as a native garden tree species and the sustainable utilization of *Rhododendron* plant resources.

## Materials and methods

### Study site and material

This research was conducted at the natural distribution site on Zhaoyun Mountain, chosen for its large population distribution and representation of all age classes of *R. platypodum*. Voucher specimens are preserved in the plant specimen room of the National Positioning Observation and Research Station of Mountainous Urban Forest Ecosystem of Chongqing (CQCSDWZ) at Chongqing Academy of Forestry Sciences. This natural population is located in Zhaoyun Mountain, Wulong District, Chongqing, near the junction of Chongqing and Guizhou Province, ranging from 29°11’40.85“to 29°12’18.61”N, between 107°26’42.72” and 107°28’44.33”E. The region experiences a subtropical humid monsoon climate with an average annual temperature of 12 °C, average annual precipitation of 1350 mm, relative humidity exceeding 80%, and about 1150 hours of annual sunshine. The soil types include mountainous yellow soil and yellow-brown soil. Covering approximately 18 ha, this population inhabits a relatively stable environment, with slops ranging from 21° to 47° and aspects facing east, south, southwest and southeast. Human disturbance in this plot is severe. *R. platypodum* primarily grows on the ridges, plains, and slopes at altitudes of 1733–1882 m. The majority of this population consists adult trees capable of flowering and fruiting with basal diameters ranging from 2 to 40 cm and ages between 10 and 300 years. Seedlings are rare, indicating that this population is overall degraded.

### Floral phenology and floral characteristics observation

The flowering dynamics, visitor activity and frequency, and effective pollination frequency of *R. platypodum* were observed in Zhaoyun Mountain. Based on the previous observation, five adult trees were randomly selected and marked in early May to monitor the flowering dynamics. Observations were conducted at a fixed times to record the start, end, and duration of the flowering period. Changes in the marked inflorescence buds, the order of flower opening, the duration of blooming, and other indicators of 20 flower buds were observed and recorded daily from 10:00 to 10:30 am. In order to document and record the visit of pollinating insects, eight flowering inflorescence were randomly selected during the flowering period on clear days. Photos were taken using a digital camera (Canon EOS R50) from 10:00 to 10:20 am and from 15:30 to 15:50 pm. Visiting insects were captured for species identification.

### Pollen Vitality and Stigma Viability Characteristics

The pollen vitality of *R. platypodum* was measured using the TTC (2,3,5-triphenyltetrazolium chloride) method [43]. Unopened buds were labeled, and samples were collected and tested every hour from 9:00 am to 19:00 pm on the second day after flowering to analyze hourly changes in pollen vitality. Daily changes in pollen vitality were documented at 9:00 am for nine consecutive days. Fresh pollen were collected and placed in a 2 mL test tube, where it was gently crushed using a dissecting needle. Then, one drop of 0.5% TTC staining solution was added to the test tube, and the samples were incubated in the dark at 37°C for 15 minutes. After incubation, the test tube was shaken to disperse the pollen evenly. The TTC staining solution containing the pollen was transferred to a glass slide using pipette, and the pollen cells were gently dispersed with a dissecting needle. Four temporary mounts were prepared using above method. Staining results were observed and photographed by a microscope (SAGA SG30). Red-stained pollen grains indicated active pollen, while unstained ones represent inactive pollen grains. In this study, five replicates were performed for pollen vitality testing.

Stigma receptivity was determined using benzidine-hydrogen peroxide method [44]. The column stigmas of *R. platypodum* at each flowering stage were placed in a 10 mL transparent plastic centrifuge tube containing benzidine-hydrogen peroxide reaction solution. The solution was prepared with a volume fraction ratio of 1% benzidine (by mass), 3% hydrogen peroxide (by volume), water in a 4:11:22 ratio. Under a steriomicroscope by observing the pistil found out, the stigma surrounded by a large number of blue bubbles around indicated strong receptivity. If no bubbles formed and stigma remained unchanged in blue, that means the stigma has no receptivity. Five replicates were conducted for stigma testing in this experiment.

The pollen ovule ratio (P/O) was determined using the standard and method of Cruden [45]. Seven buds with unopened anthers were picked from different plants. The number of pollen and ovules in each flower was counted to calculate the ratio.

### Breeding system analysis

In May 2019, the inflorescences from well-growing, sun-facing *R. platypodum* located in the upper middle of the crown were selected and treated as follows before flowering: (1) Open-pollination: In this treatment, no bagging, emasculation or artificial pollination were applied to the flower, allowing them to remain in their natural state to observe the affinity of *R. platypodum* under wild conditions. (2) Self-pollination: To test the self-compatibility of the same flower on one plant, the buds were covered with a sulfate paper bag before flowering. After flowering the bag was removed and the flower was pollinated artificially using the pollen of the same flower, and then re-enveloped again after pollination. (3) Geitonogamy (transfer of pollen to a different flower on the same plant): Flower buds were emasculated using a sterilized scissors and covered with a sulfate paper bag. On the third day of flowering, pollen of different flowers from same plant were used for artificial pollination. After pollination, the flower was re-enveloped by sulfate paper bag to test the geitonogamous pollination. (4) Xenogamy (cross-pollination): Buds were emasculated and bagged as in the geitonogamy treatment. On the third day of flowering, pollen from plants with flower opening at least 10 meters away was used for pollination. After pollination, the flower was re-enveloped with the paper bag to test for cross-compatibility. (5) Wind pollination: Flower buds were emasculated and covered with a 60-mesh bag (with an aperture of about 0.25 mm). (6) Autonomous self-pollination (cleistogamy): No emasculated flower buds were covered by sulfate paper bags to test spontaneous pollination. (7) Parthenogenesis (reproduction from an ovum without fertilization): Flower buds were covered with sulfate paper bag after emasculation without artificial pollination. The number of flowers in a single inflorescence was recorded for all seven treatments, with 20 inflorescences per treatment. On October 3, 2019, the fruits of each treatment were collected and, counted. The fruit-setting rate was calculated as follows:Fruit setting rate =Number of fruits per flower/Number of flowers accept treatment×100% .

### Statistical analysis

The data were analyzed statistically using SPSS ver. 22 (IBM SPSS Statistics for Windows). Descriptive statistics were used to calculate the means and standard deviations. One-way ANOVA was performed to analyze the differences among the hourly and daily change in pollen vitality and the artificial pollination treatments. Means were compared using Duncan’s multiple range test and the values were considered significant at p <  0.05.

## Results

### Flowering phenology period and floral traits

**Fig 1:**
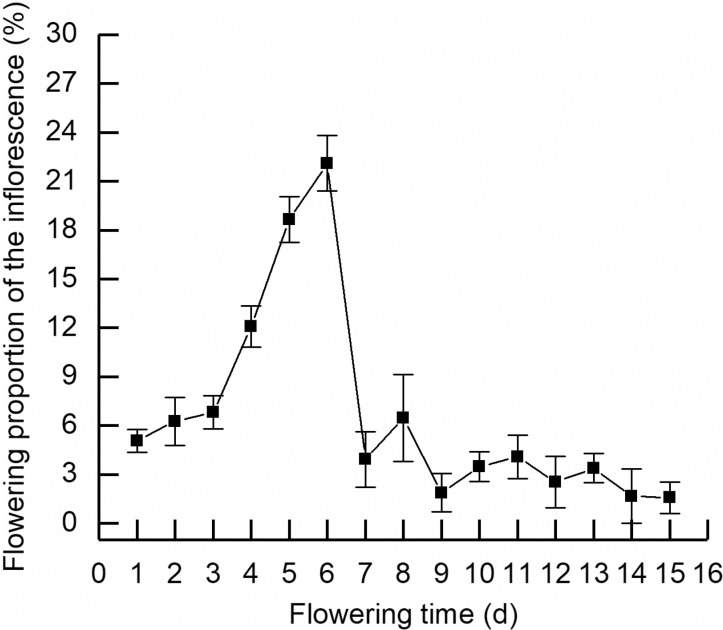
Flowering ratio of racemose umbels of *Rhododendron platypodum.*

**Fig 2:**
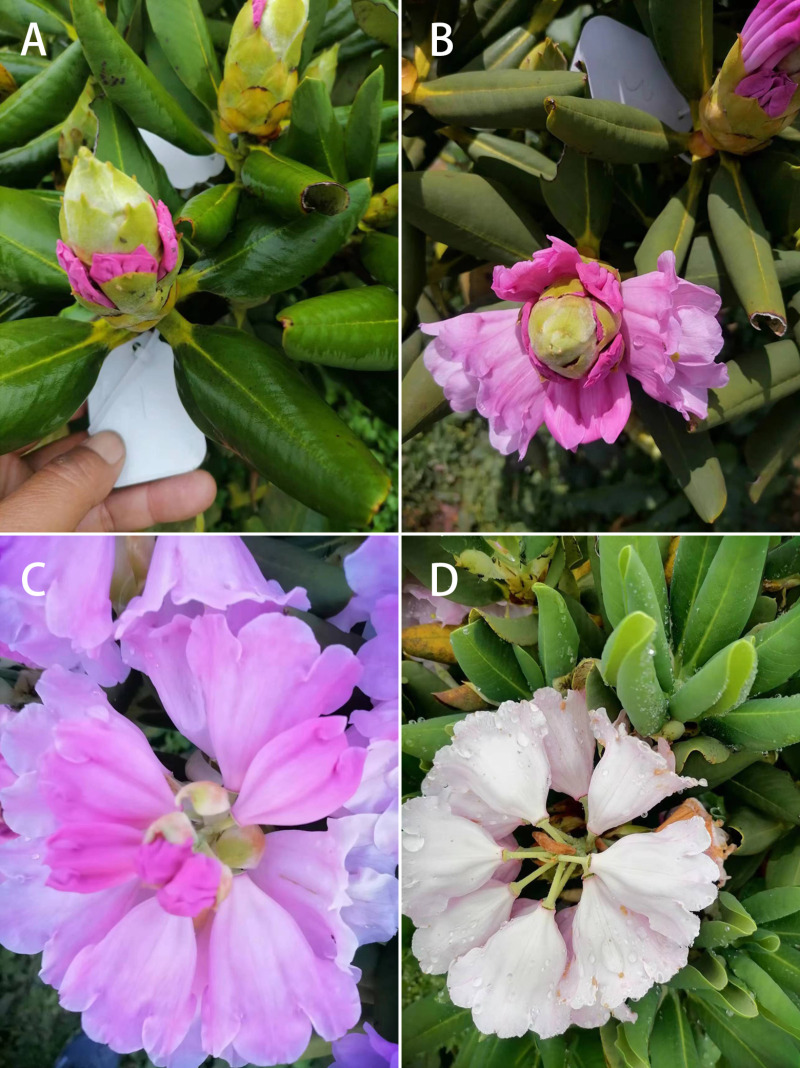
Flowering stage of racemose umbels of *Rhododendron platypodum.* A.the bud stage; B. the beginning flowering stage; C.the blooming stage; D. the fading stage.

In natural condition, the flowering period of *R. platypodum* population lasted 36 days, from mid-April to late May. The inflorescence of *R. platypodum* opened gradually from bottom to the top, with slight differences in flowering time in upper flowers due to factors such as location and light conditions. The average flowering time for a single inflorescence was about 15 days. In the early three days, the flowering proportion of the inflorescence was 6.82%. From 4th to the 6th day after emergence, the flowering entered a centralized opening phase with a flowering ratio of 12.08% on the 4th day, 18.65% on the 5th day, and 22.1% on the 6th day. From 7th to 15th day, it maintained a sporadic opening stage (Fig 1). The flowering period of a single flower lasted 9.15 days. The opening process of a single flower can be divided into bud stage, beginning flowering stage, blooming stage and fading stage (Fig 2). The leaves of *R. platypodum* were first sprout, followed by flowers and the leaf development and flowering stages overlaped partially.

The terminal inflorescence of *R. platypodum* consist of racemose umbels with an average length of (53.66 ±  0.73) mm and an average flower number of (15.07 ±  0.54). The length of light green peduncle is (33.53 ±  0.35) mm. The length of small calyx is (2.06 ±  0.07) mm. Corolla showed funnel-shaped bell, with 7 oblate lobes, pink or pinkish-red, without spots. The corolla diameter is (40.88 ±  1.48) mm, and the corolla lobe depth is (13.15 ±  0.41) mm. The number of stamens is (14.67 ±  0.21), with an average the length about (28.53 ±  0.76) mm. The white filament at the base has white soft hairs, and the anthers are light brown. The pistil has a length of (39.69 ±  0.8) mm, with a light green, disc-shaped stigma that turns brown when mature. The style is light green, and the ovary is superior, both densely covered with white glands. During the bud stage, the stigma of *R. platypodum* is enclosed by the petals. The buds are long oval, with a rose-red color. The stigma is yellowish-green and immature, secreting no mucus when the flowers are opened by tweezer (Fig 2A). At the beginning of flowering, the flowers slightly unfold, but do not reach their full bloom. The petals become light in color and the stigma begins to secrete mucus. Linear, viscous filamentous pollen overflows from the anther apical pore (Fig 2B). During the blooming period, the corolla turns pink, fully opens, and the stigma curves into an arch shape with mucus. The stamens and pistils separate, and the anthers contain the highest amount of pollen (Fig 2C). During the withering period, the corolla turns white with brown spots begin to appear on it, then corolla gradually shrinks. The stigma slightly darkens, still secreting mucus, the anthers have disperse, and the filaments begin to wither (Fig 2D).

### Pollen vitality and stigma acceptability

The pollen vitality of *R. platypodum* was highest in the morning and evening, while a significant decrease observed in the afternoon. The pollen vitality of *R. platypodum* from 9:00 to 10:00 and 16:00 to 19:00 was significantly higher than that from 13:00 to 14:00 (Table 1). Therefore, from the perspective of pollen vitality, morning and evening is more favorable for the pollination activity of *R. platypodum*.

**Table 1:** Changes of pollen viability (mean ±  SE) and stigma receptivity of *Rhododendron platypodum.* “+” indicates that the stigma has acceptability. “++” indicates that the stigma has strong acceptability. “-” indicates that the stigma is inadmissible. Different letters in column indicate significant difference, p <  0.05.

Time of the day	Pollen viability (%)	Stigma receptivity	Flowering day	Pollen viability (%)	Stigma receptivity
9:00	96.48 ± 1.28 a	+	1	60.08 ± 3.15 c	+
10:00	97.13 ± 1.43 a	+	2	83.18 ± 1.67 b	++
11:00	95.27 ± 1.29 ab	+	3	96.91 ± 0.54 a	++
12:00	94.67 ± 0.81 ab	++	4	84.83 ± 1.3 b	+
13:00	83.45 ± 2.22 c	++	5	61.96 ± 1.98 c	+
14:00	87.06 ± 2.05 bc	+	6	43.16 ± 1.73 d	–
15:00	88.95 ± 3.31 abc	+	7	18.64 ± 1.17 e	–
16:00	97.95 ± 0.59 a	+	8	4.03 ± 0.58 f	–
17:00	96.61 ± 0.88 a	+	9	0.62 ± 0.2 g	–
18:00	97.27 ± 1.32 a	++			
19:00	97.79 ± 0.87 a	–			

The pollen vitality of *R. platypodum* increased rapidly in the initial days of flowering and then declined as flowering progressed. On the first day of flowering, the pollen vitality of *R. platypodum* reached 60.08%, continued to increase on the second day, and peaked at 96.91% on the third day. Subsequently, it rapidly decreased to 4.03% by the eight day of flowering and gradually became inactive (Table 1). Therefore, the pollination time of *R. platypodum* after blooming is relatively short. We counted the days based on pollen activity higher than 50%, it was determined that effective pollination period is approximately about 5 days.

The hourly and daily changes in the stigma receptivity of *R. platypodum* are statistically summarized in Table 1. Hourly variation revealed that stigma receptivity peaked from 12:00-13:00 in the noon and around 18:00 in the afternoon of *R. platypodum*. The stigma receptivity was observed from 9:00 to11:00 and 14:00 to 17:00 but lost by 19:00. These results suggest that midday and evening periods are most favorable for pollination from the perspective of stigma acceptability in *R. platypodum*. In daily changes, stigma receptivity persisted during the first 5 days of flowering, with the strongest receptivity occurring on the second and third days, making them the most conducive to pollination.

### Hybridization index and pollen-ovule ratio

The hybridization index of *R. platypodum* was estimated by Dafni’s standard [44]. The corolla diameter was (40.88 ±  1.48) mm >  6 mm, denoted as 3. Its flower has both pistil and stamen, which mature simultaneously, denoted as 0. Additionally, the stigma is curved and positioned higher than the anther during flowering, resulting in spatial separation, denoted as 1. Therefore, the hybridization index of *R. platypodum* is 4, which can be inferred that the breeding system of *R. platypodum* is partially self-compatible, primarily cross-pollinated, and reliant on pollinators.

The average P/O value of *R. platypodum* was 750.57. According to Cruden’s [45] evaluation criteria of breeding system type, this value categorizes *R. platypodum* belongs to the facultative out-crossing breeding system.

### Breeding system analysis

**Fig 3:**
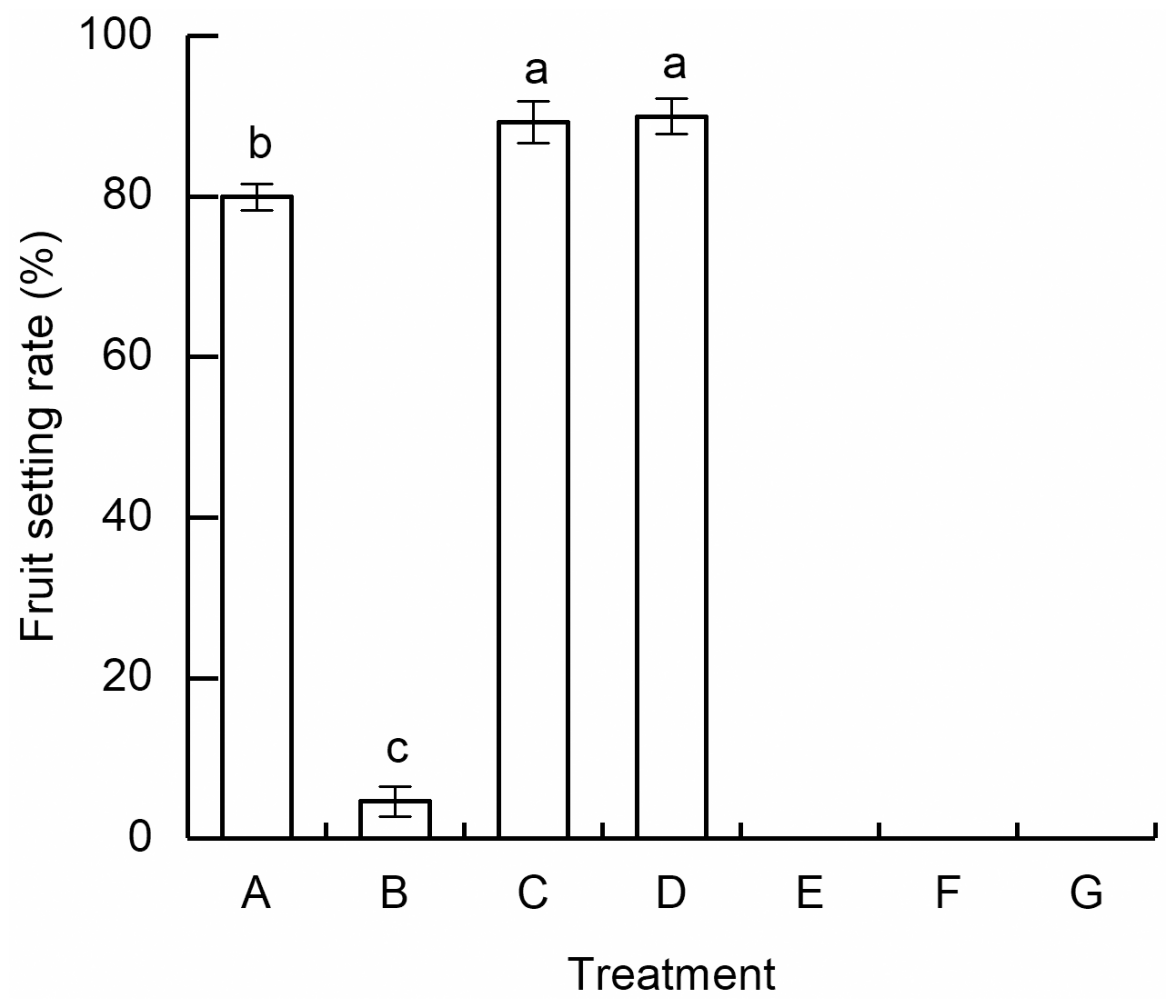
Fruit setting rate of *Rhododendron platypodum* under different treatments. A: Open-pollination. B: Self-pollination. C: Geitonogamy. D: Xenogamy. E: Wind pollination. F: Autonomous self-pollination. G: Parthenogenesis. Different letters indicate significant difference, **p** <  0.05.

According to Fig 3, the open-pollination group has a high fruit setting rate of (79.94 ±  1.61) %, significantly surpassing than that of self-pollination group, wind pollination group, insect pollination group, and single wind pollination group. The fruit setting rate of the self-pollination group was (4.63 ±  1.87)%, representing 5.79% of the open-pollination group. This indicates that while *R. platypodum* is capable of producing fruits through self-pollination, the pollination efficiency and fruit setting rate were relatively low. classifying it as a facultative heterozygous species. The wind pollination group, autonomous self-fertilization, and single wind pollination group did not produce seeds, indicating that the pollination process of *R. platypodum* could not be completed solely by wind pollination and required assistance of insects or humans in this process. The seed setting rates of geitonogamy group and xenogamy groups were significantly higher than in the open-pollination group, reaching (89.23 ±  2.6) % and (89.98 ±  2.21) %, respectively (Fig 3). This suggests that artificial pollination can further enhance the pollination efficiency of *R. platypodum* compared to natural pollination.

### Visiting Insects

**Fig 4:**
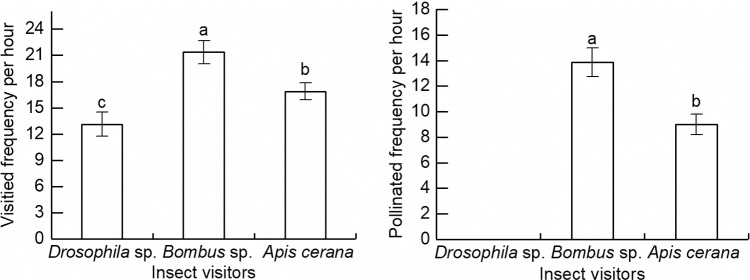
Main pollinators and single inflorescence pollination of *Rhododendron platypodum.* Different letters indicate significant difference, **p** <  0.05.

The main pollinating insects of *R. platypodum* were identified as *Bombus* sp., *Apis cerana*, and *Drosophila* sp. Statistical analysis of camera records revealed that *Bombus* sp. had the highest average visiting frequency of a single inflorescence, reaching (21.38 ±  1.32) times per hour, which was significantly higher by 1.63 and 1.27 times compared to *A. cerana* and *Drosophila* sp., respectively. The visiting frequency of *A. cerana* (16.88 ±  0.97 times/h) was significantly lower than that of *Bombus* sp. but significantly higher than that of *Drosophila* sp. (13.13 ±  1.38 times/h). Among the three insect groups, *Bombus* sp. also demonstrated the highest effective pollination frequency (13.88 ±  1.13 times/h), which is significantly higher than that of *A. cerana* and *Drosophila* sp. The effective pollination frequency of *Bombus* sp. was 1.54 times higher than *A. cerana*, while no effective pollination frequency was observed for *Drosophila* sp. (Fig 4).

## Discussion

Previous studies have found that the natural habitat of *R. platypodum* is characterized by shallow soil layers, poor soil fertility, poor site conditions, fragmented habitats, sparse seedlings, slow and difficult population regeneration [37, 38], all of which contribute to its gradually endangered status [36]. Factors leading to endangerment of plants mainly includes internal attributes and external interference, with reproductive system disorders being a key internal factor [9]. Researching on the flowering biology and breeding system of *R. platypodum* is therefore critical for understanding its endangerment mechanisms and informing future conservation strategies.

Floral traits (including flower color, structure, aroma, etc.) and opening pattern (flower size, number of flowers, etc.) play a significant role in attracting pollinators, as well as influencing the efficiency of self- and cross- pollination, ultimately affecting pollination success [2,46,47]. Wang et al. [48] found that pollinators favor inflorescences over individual flowers. In this study, *R. platypodum* was observed to have large corolla, with fragrance free pink flowers, non-folding petals and high floral density during its flowering period. These traits coupled with the presence of nectaries, classify it as an entomophilous flower [49–50], attracting pollinator such as *Bombus* and *Apis cerana* [2,51]. Flower traits can evolve to adapt to distinct pollinators [52–54], with large flowers and low pigment content (i.e., anthocyanin and carotenoid) being particularly attractive to bees [55]. The cylindrical, funnel-shaped corolla and shallow color of *R. platypodum* is well-adapted to the body structure and preference of (bumble)bees. Field observations revealed that flies only briefly stayed at the edge of the corolla of *R. platypodum* (4–7 seconds without contacting with the stamens and pistils, whereas (bumble)bees burrowed into the interior of flowers, and stayed for a slightly longer period of time to collect pollen. During this process, pollen adhere to their feet and abdomen, which was subsequently transferred to the stigma of other flowers, facilitating cross-pollination. The terminal raceme umbel of *R. platypodum* with its dense floral arrangements, the average anthesis of inflorescence duration was (13.4 ±  0.75) days, which facilitates the movement of pollinators between different inflorescences, making self- or cross- pollination within the same plant inevitable. This aligns with findings on *R. machongensis* [28]. Additionally, studies on *R. ferrugineum* have shown the absence of pollinators, long stamen do not facilitate self-pollination, while short stamen provide reproductive guarantee for the population [22]. *Rhododendron* species possess stamens of varying lengths, and their role in reproduction remains to be further studied.

Floral display significantly influences pollen dispersal patterns through its attraction to pollinators, which in turn affects the mating system of plants [56]. In general, floral display with more flowers are more conspicuous than those with fewer flowers, thereby attracting a higher number of pollinators [57]. In this study, *R. platypodum* exhibited synanthesis, with the pistil and stamen maturing simultaneously—a characteristics also observed in *Rhododendron siderophyllum* and *Rhododendron fortunei* [26,58]. Most rhododendrons exhibit diplogamy [24], indicating the possibility of self-pollination, which has also been confirmed in artificial pollination experiments (Fig 3). The pollen viability of *R. platypodum* peaked 96.91% on the third day of flowering, which was higher than that of existing rhododendrons [21,31,59]. However, its pollen vitality remained above 50% for only 5 days, and dropped significantly after 7 days, indicating high initial vitality but a short viability period in *R. platypodum*, which is unfavorable to the pollination success of this species. Moreover, some studies found that the pollination activity of European bees in apple orchards correlates significantly with local temperature, solar radiation and wind speed [60]. In present study, the site experienced frequent rain (29 days during flowering period), accompanied by cooling and strong winds, which may also affect the activity frequency of pollinating insects [61], thereby affecting the pollination efficiency of *R. platypodum*. When the stigma receptivity was at its peak, pollen activity was also highest, indicating the co-maturation of stamen and pistil of *R. platypodum*. However, during this period, the style of *R. platypodum* was curved and arched, with the stigmas positioned higher than the stamens, resulting in spatial ectopic (herkogamy) phenomenon that helps bisexual plants effectively avoid self-mating [62]. Such spatial separation accommodates spatial locations accommodate different pollinators, promoting effective pollination. For instance, de Vos et al. [63] found a separation greater than 1 mm between stamens and stigma in mature *Primula halleri* flowers favors outbreeding. In our study on *R. platypodum*, the length difference between stamens and stigma was 11.16 mm, significantly exceeding 1 mm, strongly suggesting a preference for outbreeding [64]. This phenomenon has also been confirmed in the experiment of autonomous self-pollination with resulting of no fruit be set. In subsequent breeding, pollen can be collected at 16:00 on the third day of flowering for pollination, or the stigma of 2-3 days after flowering can be pollinated at 12:00-13:00, which may improve pollination efficiency and setting more fruits.

Based on the calculation of outcrossing index (OCI) and pollen/ovule ratio (P/O), the breeding system of *R. platypodum* classified as facultative outcross type, self-compatible, and sometimes requires pollinators [44,45], aligning with the breeding systems of *R. siderophyllum* [26], *R. maxiongense* [28], and *R. faberi* [29]. The P/O ratio, an indicator of male resource allocation, was 750.57 in this study, closely aligned with values indicative of facultative xenogamy [45]. This suggests that the breeding system of *R. platypodum* leans more towards an outbreeding system, consistent with findings on endemic and endangered plant of *Opisthopappus longilobus* and *Opisthopappus taihangensis* grown at cliff habitat [65].

In order to accurately determine the breeding system of *R. platypodum*, artificial pollination tests were conducted to enhance the reliability of findings [66]. The absence of fruit set in bagging treatment without emasculation and bagging with emasculation treatment, indicating that *R. platypodum* does not undergo automatic self-pollination or parthenogenesis. Fruit set was observed in both artificial self-pollination and artificial cross-pollination treatments, confirming that *R. platypodum* is compatible with both self- and cross- pollination, similar to *R. longipedunculus*, *R. ferrugineum* and *R. vialii* [28,33]. Additionally, the fruit setting rates in these artificial self-pollination and artificial cross-pollination treatments were higher than those observed under natural pollination, suggesting that *R. platypodum* experiences pollen restriction [67–70], a common trait among *Rhododendrons* [21,31]. Pollen limitation also affects the reproduction of other endangered shrub, such as *Lonicera oblata* at cliff habitats [71]. The absence of fruit set from wind pollination indicates that *R. platypodum* mainly depended on insect pollinator, consistent with studies showing that *Rhododendrons* are predominantly insect-pollinated [72,73]. Observations during flowering revealed that *R. platypodum* exhibits an arched stigma, similar to *Rhododendron aureum* and *R. longipedunculus* [33,74], which effectively prevents self-pollination and supports a mixed mating system dominated by outcrossing. There was no difference in fruit setting rate was found between artificial geitonogamy and artificial xenogamy treatments. Meanwhile, field observation showed that pollinators often visited adjacent inflorescences on the same plant, indicating that the main pollination mode of this species under natural conditions was geitonogamy, as observed in many *Rhododendrons* pollinate [23,25,72,75]. Inbreeding in plants often induced by population size and distribution [76,77]. While inbreeding can lead to population decline and reduce offspring fitness [78], it is frequently considered as a reproductive security mechanism, especially under conditions of pollen restriction [79]. Although the population of *R. platypodum* is small, the SSR studies indicate no significant inbreeding within its population [80]. Field investigations revealed rare seedlings within the population [37,38], potentially due to reduced seed germination rates or declined seedling fitness from inbreeding, or unsuitable germination conditions. However, we did not conduct any seed germination and seedling growth experiments representing limitation of our study that need for further research.

The mutualistic cooperation between flowering plants and pollinators is shaped by long-term co-evolution [81,82]. Pollination efficiency varies based on the behaviors of visiting insects [72,83]. Floral-visiting insects of *Rhododendrons* are relatively diverse, involving 4 orders and 16 families according to statistics [84], yet bumblebees are widely recognized as their effective pollinators [23,25,85]. At the same time, many *Rhododendrons* have specialized pollinators [73,84]. The low frequency ultrasonic waves generated by bumblebee wing movement during nectar collection facilitate pollen release from stamens in *Rhododendron maddenii* and *Rhododendron augustini* [86]. Unlike flies, ants, and beetles, bumblebees can carry and collect large quantities of linear viscous pollen [72]. Many studies have shown that bumblebees are the most important pollinators of the groups with poricidally dehiscent anthers among temperate plants, including *Rhododendrons* [25,67,72,75,87,88]. In this study, bumblebees, *A. cerana* and *Drosophila* were the main flower visitors of *R. platypodum*. Among them, both bumblebee (*B. braccatus*) and Chinese bee (*A. cerana*) carried pollen and actively transferred pollen to stigma when visiting flowers. While drosophila only fed on nectar and did not transfer pollen to stigma. Therefore, bumblebee and Chinese bee are effective pollinators of *R. platypodum*.

## Conclusion

The floral features of *R. platypodum* showed adaptability to insect pollination. At both the individual and population levels, *R. platypodum* exhibits higher flowering synchronicity, which attracts more visitors and produce higher capsule setting rate. The spatial ectopic of stamens and pistils could avoid self-cross to a certain extent. The breeding system of *R. platypodum* is a mixed mating system with preference for outcrossing, and its effective pollinators under natural conditions are *Bombus* sp. and *A. cerana*. The wild population of *R. platypodum* on Zhaoyun Mountain is located in the tourism development zone, subject to significant human disturbance. Meanwhile, pollen restriction in populations, windy and rainy weather on flowering periods, will affect the seed production of *R. platypodum*. Moreover, high geitonogamy relying on pollinators for effective production revealed by artificial pollination is likely to lead to a decrease in the adaptability of the offspring of this species. To expand population of *R. platypodum,* artificial breeding can be employed, along with efforts to increase outcrossing probabilities under natural conditions by releasing effective insects during the flowering period. While rainfall exacerbates flowers and fruits drop, field observations show that *R. platypodum* begins to bloom at around 7–8 years of age, with flowering intensity increasing as the tree ages. Remarkably, this species continue blooming abundantly for centuries, with some trees blooming even at 250 years of age. Each capsule contains hundreds of seeds [89,90] and the 80% capsule setting rate in natural pollination in this study ensures a reliable amounts of seeds. Therefore, alongside mitigating human interference, it is crucial to focus attention on seed dispersal, germination, and the environmental requirements for growth of seedlings and saplings of *R. platypodum*.

## References

[1] Daniels R, Jayanthi M. Biology and conservation of endangered plants: The need to study breeding systems. Tropical Ecology. 1996;37:39–42.

[2] Ågren J. Pollinators, herbivores, and the evolution of floral traits. Science. 2019;364(6436):122–3. doi: 10.1126/science.aax1656 30975872

[3] Grant V. Plant speciation. 2nd ed. New York: Columbia University Press; 1981. doi: 10.7312/gran92318-037

[4] Wyatt R. Pollinator–Plant Interactions and the Evolution of Breeding Systems. Pollination Biology. 1983;51–95. doi: 10.1016/b978-0-12-583980-8.50011-9

[5] Mitchell R, Karron J, Holmquist K, Bell J. The influence of Mimulus ringens floral display size on pollinator visitation patterns. Functional Ecology. 2004;18:116–24. https://www.jstor.org/stable/3599013

[6] Charlesworth D. Evolution of plant breeding systems. Curr Biol. 2006;16(17):R726-35. doi: 10.1016/j.cub.2006.07.068 16950099

[7] Zhang Q, Zhang H, Sun L, Fan G, Ye M, Jiang L, et al. The genetic architecture of floral traits in the woody plant *Prunus mume*. Nat Commun. 2018;9(1):1702. doi: 10.1038/s41467-018-04093-z 29703940 PMC5923208

[8] Sapir Y, Brunet J, Byers DL, Imbert E, Schönenberger J, Staedler Y. Floral Evolution: Breeding Systems, Pollinators, and Beyond. International Journal of Plant Sciences. 2019;180(9):929–33. doi: 10.1086/706240

[9] Huang Z. The research progress of endangered causes and protection strategy of rare and endangered plants in China. Journal of University of South China (Science and Technology). n.d.;34 42–50. doi: 10.19431/j.cnki.1673-0062.2020.03.007

[10] Barrett SCH, Eckert CG. Variation and Evolution of Mating Systems in Seed Plants. Biological Approaches and Evolutionary Trends in Plants. 1990 229–54. doi: 10.1016/b978-0-12-402960-6.50019-6

[11] Naiki A. Heterostyly and the possibility of its breakdown by polyploidization. Plant Species Biology. 2012;27(1):3–29. doi: 10.1111/j.1442-1984.2011.00363.x

[12] Paź-Dyderska S, Jagodziński AM. Potential of reproductive traits in functional ecology: A quantitative comparison of variability in floral, fruit, and leaf traits. Ecol Evol. 2024;14(7):e11690. doi: 10.1002/ece3.11690 39026952 PMC11255459

[13] Simón-Porcar V, Escudero M, Santos-Gally R, Sauquet H, Schönenberger J, Johnson SD, et al. Convergent evolutionary patterns of heterostyly across angiosperms support the pollination-precision hypothesis. Nat Commun. 2024;15(1):1237. doi: 10.1038/s41467-024-45118-0 38336937 PMC10858259

[14] Chen X, Lu X, Liu Y, Zhao M, Cui X, Zhang D. Floral morphology and flowering process of *Acer yangjuechi*, the extremely endangered plant. Bulletin of Botanical Research. 2019;39 329–37. doi: 10.7525/j.issn.1673-5102.2019.03.002

[15] Chen Z, Wei X, Chai S, Zou R, Wang J. Studies on the flowering and fruiting phenology and mating system of the endangered plant *Sauvagesia rhodoleuca*. Guangxi Sciences. n.d.;30 1091–100. doi: 10.13656/j.cnki.gxkx.20240125.007

[16] Guo L, Lin G, Xu W, Wang A. Characteristics of reproductive modules of *Emmenopterys henryi* natural population in Wuyi mountain. Journal of Northwest Forestry University. 2011;26(1):18–22.

[17] Wang Q. Conservation strategies of a Chinese native tree species: yellowhorn (*Xanthoceras sorbifolium*) based on conservation biology. PhD. Thesis, Beijing Forestry University. 2019. Available from: doi: 10.26949/d.cnki.gblyu.2019.000012

[18] Fu G, Li T, Zhang Y, Wen X, Ma X, Wu C. Phenological observation of the extremely small population plant *Styrax zhejiangensis*. Bulletin of Botanical Research. 2021;41(2):168–73. doi: 10.7525/j.issn.1673-5102.2021.02.003

[19] Bhatnagar S, Kumari R. Floral biology, pollination biology, and breeding mechanisms reveal challenges to the restoration of *Buchanania lanzan*, a vulnerable plant species. Flora. 2024;311:152448. doi: 10.1016/j.flora.2024.152448

[20] Grave E, Kroessig T, Ticktin T. Pollination biology of an endemic Hawaiian tree, *Erythrina sandwicensis* (Fabaceae: Papilionoideae), in a novel ecosystem. Pacific Science. 2021;75(3):289–308. doi: 10.2984/75.3.1

[21] Xie M, Ma Y, Cao Y, Liu D, Li Z, Ma H. Research on floral syndrome and breeding systems of *Rhododendron hemsleyanum*, a plant species with extremely small populations. Journal of Yunnan Agricultural University (Natural Science). 2023;38:95–103. doi: 10.12101/j.issn.1004-390X(n).202207033

[22] Escaravage N, Flubacker E, Pornon A, Doche B, Till‐Bottraud I. Stamen dimorphism in *Rhododendron ferrugineum* (Ericaceae): development and function. American J of Botany. 2001;88(1):68–75. doi: 10.2307/2657128 11159128

[23] Stout J. Pollination of invasive *Rhododendron ponticum* (Ericaceae) in Ireland. Apidologie. 2007;38(2):198–206. doi: 10.1051/apido:2006071

[24] STOUT JC. Reproductive biology of the invasive exotic shrub, *Rhododendron ponticum* L. (Ericaceae). Botanical Journal of the Linnean Society. 2007;155(3):373–81. doi: 10.1111/j.1095-8339.2007.00719.x

[25] Ono A, Dohzono I, Sugawara T. Bumblebee pollination and reproductive biology of *Rhododendron semibarbatum* (Ericaceae). J Plant Res. 2008;121(3):319–27. doi: 10.1007/s10265-008-0155-y 18392555

[26] Bai T, Guan W, Song J, Xie W, Li S. Flowering characteristics and breeding system of *Rhododendron siderophyllum*. Journal of West China Forestry Science. 2014;43:47–53. doi: 10.3969/j.issn.1672-8246.2014.01.009

[27] Liu Q, Yi C, Zheng S, Chen K, Gu K, Zhang J. Pollination biology of vulnerable *Rhododendron vialii* (Ericaceae) in Yunnan province. Journal of West China Forestry Science. 2017;46:96–102. doi: 10.16473/j.cnki.xblykx1972.2017.03.016

[28] Yi C, Zheng S, Liu Q, Gu K, Liu G, Zhang J. Preliminary study on the breeding system and crossbreeding of *Rhododendron maxiongense* (Ericaceae). Journal of Yunnan Agricultural University (Natural Science). 2018;33:99–105. doi: 10.12101/j.issn.1004-390X(n).201611062

[29] Yu H, Wei S, Jiang X, Liu Y, Liao C, Ji H. Study on the breeding system of *Rhododendron prattii* Franch. Journal of Sichuan Forestry Science Technology. 2021;42:40–7. doi: 10.12172/202108110001

[30] Sorokhaibam SS, Chandra A, Baishya R, Barik SK, Goel S, Tandon R. Contradistinctive floral attributes, pollination guilds and their consequence on the outcrossing rate in two elevational morphs of *Rhododendron arboreum* Sm. Front Plant Sci. 2024;15:1355680. doi: 10.3389/fpls.2024.1355680 38606073 PMC11007036

[31] Xiong W, Wu Y, Bai T, Xie W, Dong Y, Zhang J. Breeding system and hybridization affinity of *Rhododendron irroratum*. Bulletin of Botanical Research. 2024;44:370–9. doi: 10.7525/j.issn.1673-5102.2024.03.006

[32] Zhang X. Reproductive biology and conservation genetics of *Rhododendron* *sinofalconeri*. M.Sc. Thesis, Chinese Academy of Forestry. 2020. Available from: doi: 10.27625/d.cnki.gzlky.2020.000141

[33] Li T, Liu X, Li Z, Ma H, Wan Y, Liu X, et al. Study on Reproductive Biology of *Rhododendron longipedicellatum*: A Newly Discovered and Special Threatened Plant Surviving in Limestone Habitat in Southeast Yunnan, China. Front Plant Sci. 2018;9:33. doi: 10.3389/fpls.2018.00033 29445383 PMC5797782

[34] Ministry of Environmental Protection, Chinese Academy of Sciences. China species red list: Higher plants. 2013 Sep 2 [cited 14 January 2025]. In: Ministry of Ecology and Environment of the People’s Republic of China [Internet]. Beijing: Ministry of Ecology and Environment of the People’s Republic of China 2018 -. [about 1 screens]. Available from: https://www.mee.gov.cn/gkml/hbb/bgg/201309/t20130912_260061.htm

[35] Gibbs D, Chamberlain D, Argent G. The red list of Rhododendrons. Botanic Gardens Conservation International. 2011. Available from: https://www.bgci.org/files/Worldwide/Publications/PDFs/RhododendronsRedList-lowRes.pdf

[36] Wang G, Deng H, Li Y, Wang Q, Zong X, Yang X. An IUCN criteria-based assessment of the endangered categories of key protected wild plants in Chongqing municipality. Journal of Southeast University (Natural Science Edition). 2017;39 42–50. doi: 10.13718/j.cnki.xdzk.2017.09.007

[37] Wang Z, Ma L, Wang H. Population characteristics of *Rhododendron platypodum* and its relationship with environmental factors in Chongqing city. Journal of Plant Resources and Environment. 2022;31(1):61–8. doi: 10.3969/j.issn.1674-7895.2022.01.08

[38] Huang M, Dai X, Yang C, Deng L, Yuan C, Li H, et al. Characteristics and diversity of typical *Rhododendron platypodum* communities in Guizhou. Journal of West China Forestry Science. 2020;49 113–20. doi: 10.16473/j.cnki.xblykx1972.2020.02.017

[39] Levin S, Muller-Landau H, Nathan R, Chave J. The ecology and evolution of seed dispersal: a theoretical perspective. Annual Review of Ecology, Evolution, and Systematics. 2003;34:575–604. doi: 10.1146/annurev.ecolsys.34.011802.132428

[40] Bittencourt JVM, Sebbenn AM. Patterns of pollen and seed dispersal in a small, fragmented population of the wind-pollinated tree *Araucaria angustifolia* in southern Brazil. Heredity (Edinb). 2007;99(6):580–91. doi: 10.1038/sj.hdy.6801019 17928810

[41] Neuschulz EL, Mueller T, Schleuning M, Böhning-Gaese K. Pollination and seed dispersal are the most threatened processes of plant regeneration. Sci Rep. 2016;6:29839. doi: 10.1038/srep29839 27435026 PMC4951728

[42] Jordano P. Fruits and frugivory. In: Fenner M, editor. Seeds: the ecology of regeneration in plant communities, 2nd ed. CABI Publishing; 2022. pp. 125−166. doi: 10.1079/9780851994321.0125

[43] Xu H, Wang Y. Study on pollination biology of Chinese medicinal material *Corydalis yanhusuo* W. T. Wang. Medicinal Plant. 2012;9:10–2.

[44] Dafni A. Pollination ecology: a practical approach. Oxford: Oxford University Press; 1992.

[45] Cruden RW. Pollen-ovule ratios: a conservative indicator of breeding systems in flowering plants. Evolution. 1977;31(1):32–46. doi: 10.1111/j.1558-5646.1977.tb00979.x 28567723

[46] Barrett SC, Harder LD. Ecology and evolution of plant mating. Trends Ecol Evol. 1996;11(2):73–9. doi: 10.1016/0169-5347(96)81046-9 21237765

[47] Fornoff F, Klein A, Hartig F, Benadi G, Venjakob C, Schaefer HM, et al. Functional flower traits and their diversity drive pollinator visitation. Oikos. 2017;126(7):1020–30. doi: 10.1111/oik.03869

[48] Wang H, Ran N, Jiang H-Q, Wang Q-Q, Ye M, Bowler PA, et al. Complex floral traits shape pollinator attraction to flowering plants in urban greenspaces. Urban Forestry & Urban Greening. 2024;91:128165. doi: 10.1016/j.ufug.2023.128165

[49] Herrera J. Visibility vs. biomass in flowers: exploring corolla allocation in Mediterranean entomophilous plants. Ann Bot. 2009;103(7):1119–27. doi: 10.1093/aob/mcp046 19258340 PMC2707908

[50] Walas Ł, Mandryk W, Thomas PA, Tyrała-Wierucka Ż, Iszkuło G. Sexual systems in gymnosperms: A review. Basic and Applied Ecology. 2018;31:1–9. doi: 10.1016/j.baae.2018.05.009

[51] Whitney HM, Glover BJ. Morphology and development of floral features recognised by pollinators. Arthropod-Plant Interactions. 2007;1(3):147–58. doi: 10.1007/s11829-007-9014-3

[52] Junker R, Blüthgen N, Brehm T, Binkenstein J, Paulus J, Schaefer H. Specialization on traits as basis for the niche-breadth of flower visitors and as structuring mechanism of ecological networks. Functional Ecology. 2013;27(3):329–41. doi: 10.1111/1365-2435.12005

[53] Dellinger AS, Artuso S, Pamperl S, Michelangeli FA, Penneys DS, Fernández-Fernández DM, et al. Modularity increases rate of floral evolution and adaptive success for functionally specialized pollination systems. Commun Biol. 2019; 2: 453. doi: 10.1038/s42003-019-0697-7 31872071 PMC6895197

[54] Basnett S, Krpan J, Espíndola A. Floral traits and their connection with pollinators and climate. Ann Bot. 2025;135(1–2):125–40. doi: 10.1093/aob/mcae046 38502826 PMC11805930

[55] Schemske DW, Bradshaw HD Jr. Pollinator preference and the evolution of floral traits in monkeyflowers (Mimulus). Proc Natl Acad Sci U S A. 1999;96(21):11910–5. doi: 10.1073/pnas.96.21.11910 10518550 PMC18386

[56] Goodwillie C, Sargent RD, Eckert CG, Elle E, Geber MA, Johnston MO, et al. Correlated evolution of mating system and floral display traits in flowering plants and its implications for the distribution of mating system variation. New Phytol. 2010;185(1):311–21. doi: 10.1111/j.1469-8137.2009.03043.x 19807872

[57] Makino T, Ohashi K, Sakai S. How do floral display size and the density of surrounding flowers influence the likelihood of bumble bee revisitation to a plant?. Functional Ecology. 2007;21 87–95. doi: 10.1111/j.1365-2435.2006.01211.x

[58] Bian C, Jin Z. Studies on floral dynamic and breeding system of *Rhododendron* *fortunei*. Guihaia. 2005;25(2):169–73. doi: 10.3969/j.issn.1000-3142.2005.02.020

[59] Qiu J, Gao C, Wei H, Wang B, Hu Y, Guo Z. Flowering biology of *Rhododendron pulchrum*. Horticulturae. 2021;7(11):508. doi: 10.3390/horticulturae7110508

[60] Vicens N, Bosch J. Weather-dependent pollinator activity in an apple orchard, with special reference to *Osmia cornuta* and *Apis mellifera* (Hymenoptera: Megachilidae and Apidae). Environmental Entomology. 2000;29(3):413–20. doi: 10.1603/0046-225X-29.3.413

[61] Carvallo GO, Medel R. Effects of herkogamy and inbreeding on the mating system of *Mimulus luteus* in the absence of pollinators. Evolutionary Ecology. 2010;24:509–22. doi: 10.1007/s10682-009-9322-4

[62] de Vos JM, Keller B, Isham ST, Kelso S, Conti E. Reproductive implications of herkogamy in homostylous primroses: variation during anthesis and reproductive assurance in alpine environments. Functional Ecology. 2012;26(4):854–65. doi: 10.1111/j.1365-2435.2012.02016.x

[63] de Vos JM, Keller B, Zhang L-R, Nowak MD, Conti E. Mixed Mating in Homostylous Species: Genetic and Experimental Evidence from an Alpine Plant with Variable Herkogamy, *Primula halleri*. International Journal of Plant Sciences. 2018;179(2):87–99. doi: 10.1086/695527

[64] Webb CJ, Lloyd DG. The avoidance of interference between the presentation of pollen and stigmas in angiosperms II. Herkogamy. New Zealand Journal of Botany. 1986;24(1):163–78. doi: 10.1080/0028825x.1986.10409726

[65] Wang Y, Lan Y, Ye H, Feng X, Qie Q, Liu L, et al. Reproductive Biology and Breeding Systems of Two Opisthopappus Endemic and Endangered Species on the Taihang Mountains. Plants (Basel). 2023;12(10):1954. doi: 10.3390/plants12101954 37653873 PMC10222883

[66] Acquaah G. Conventional plant breeding principles and techniques. In: Al-Khayri J, Jain S, Johnson D, editors. Advances in plant breeding strategies: breeding, biotechnology and molecular tools. Cham: Springer, Cham; 2015. pp. 115–158. doi: 10.1007/978-3-319-22521-0_5

[67] Kearns CA, Inouye DW, Waser NM. ENDANGERED Mutualisms: The Conservation of Plant-Pollinator Interactions. Annu Rev Ecol Syst. 1998;29(1):83–112. doi: 10.1146/annurev.ecolsys.29.1.83

[68] Basnett S, Ganesan R, Devy SM. Floral traits determine pollinator visitation in *Rhododendron* species across an elevation gradient in the Sikkim Himalaya. Alp Botany. 2019;129(2):81–94. doi: 10.1007/s00035-019-00225-3

[69] Roy SK, Khanduri P, Bhatnagar AK, Pandey AK. Pollination biology and breeding system analysis of *Ulmus wallichiana* Planchon (Ulmaceae), a rare and threatened tree species of Central and Western Himalaya. Nordic Journal of Botany. 2021;39(6):. doi: 10.1111/njb.02976

[70] Su ZH, Zhuo L, Min XJ, Wang XY. Breeding system and pollination ecology of an endangered desert shrub endemic to northwestern China. Nordic Journal of Botany. 2024;2024(5):. doi: 10.1111/njb.04079

[71] Wu Y-M, Shen X-L, Tong L, Lei F-W, Xia X-F, Mu X-Y, et al. Reproductive biology of an endangered lithophytic shrub and implications for its conservation. BMC Plant Biol. 2022;22(1):80. doi: 10.1186/s12870-022-03466-3 35193519 PMC8862588

[72] Escaravage N, Wagner J. Pollination effectiveness and pollen dispersal in a *Rhododendron ferrugineum* (Ericaceae) population. Plant Biol (Stuttg). 2004;6(5):606–15. doi: 10.1055/s-2004-821143 15375732

[73] Hirao AS, Kameyama Y, Ohara M, Isagi Y, Kudo G. Seasonal changes in pollinator activity influence pollen dispersal and seed production of the alpine shrub *Rhododendron aureum* (Ericaceae). Mol Ecol. 2006;15(4):1165–73. doi: 10.1111/j.1365-294X.2006.02853.x 16599975

[74] Kudo G. Relationships between flowering time and fruit set of the entomophilous alpine shrub, *Rhododendron aureum* (Ericaceae), inhabiting snow patches. American Journal of Botany. 1993;80:1300–4. doi: 10.1002/j.1537-2197.1993.tb15368.x

[75] Ma YP, Wu ZK, Dong K, Sun WB, Marczewski T. Pollination biology of *Rhododendron cyanocarpum* (Ericaceae): An alpine species endemic to NW Yunnan, China. Journal of Systematics and Evolution. 2015;53:63–71. doi: 10.1111/jse.12114

[76] Qiao Q, Zhang C, Milne R. Population genetics and breeding system of *Tupistra pingbianensis* (Liliaceae), a naturally rare plant endemic to SW China. Journal of Systematics and Evolution. 2010;48:47–57. doi: 10.1111/j.1759-6831.2009.00064.x

[77] Martínez-García PJ, Dicenta F, Ortega E. Anomalous embryo sac development and fruit abortion caused by inbreeding depression in almond (*Prunus dulcis*). Scientia Horticulturae. 2012;133:23–30. doi: 10.1016/j.scienta.2011.10.001

[78] Ng S, Corlett R. Comparative reproductive biology of the six species of *Rhododendron* (Ericaceae) in Hong Kong, South China. Canadian Journal of Botany. 2000;78:221–9. doi: 10.1139/b99-181

[79] Liu S, Wu L, Huang S. Shortened anther–stigma distance reduces compatible pollination in two distylous *Primula* species. Journal of Plant Ecology. 2016;9 224–32. doi: 10.1093/jpe/rtv049

[80] Ma L, Li L, Fang W, Dong Z, Liu Y, Wang C. Genetic variability and population divergence of *Rhododendron platypodum* Diels in China in the context of conservation. Frontiers in Forests and Global Change. 2024;7:1320995. doi: 10.3389/ffgc.2024.1320995

[81] Nicolson S, Wright G. Plant–pollinator interactions and threats to pollination: perspectives from the flower to the landscape. Functional Ecology. 2017;31(1):22–5. doi: 10.1111/1365-2435.12810

[82] Sun S, Leshowitz MI, Rychtář J. The signalling game between plants and pollinators. Sci Rep. 2018;8(1):6686. doi: 10.1038/s41598-018-24779-0 29703897 PMC5923245

[83] Pan C, Chen Z, Zhang M, Chen X, Smagghe G, Fan M, et al. Effects of flowering period on floral traits, pollinator behavior and seed production of David’s mountain laurel (*Sophora davidii*). Plant Signal Behav. 2024;19(1):2383823. doi: 10.1080/15592324.2024.2383823 39066647 PMC11285235

[84] Tian X, Ma Y, Zhang C, Tang G, Zhang J. Recent advance of reproductive biology in *Rhododendron* L. Journal of Nanjing Forestry University (Natural Science Edition). 2011;35(3):124–8. doi: 10.3969/j.jssn.1000-2006.2011.03.026

[85] Fenster C, Armbruster W, Wilson P, Dudash M, Thomson J. Pollination syndromes and floral specialization. Annual Review of Ecology, Evolution, and Systematics. 2004;35:375–403. doi: 10.1146/annurev.ecolsys.34.011802.132347

[86] King MJ, Buchmann SL. Bumble bee-initiated vibration release mechanism of *Rhododendron* pollen. American Journal of Botany. 1995;82:1407–11. doi: 10.2307/2445867

[87] Kudo G, Hirao A, Kawai Y. Pollination efficiency of bumblebee queens and workers in the alpine shrub *Rhododendron aureum*. International Journal of Plant Sciences. 2011;172:70–7. doi: 10.1086/657282

[88] Takahashi K, Itino T. Visitation frequencies of bumblebees and swallowtail butterflies to flowers and the nectar sugar concentration of *Rhododendron kaempferi* and *R. japonicum* in mountains of central Japan. Journal of Pollination Ecology. 2017;21:92–7. doi: 10.26786/1920-7603(2017)438

[89] Williams E, Kaul V, Rouse J, Knox R. Apparent self-incompatibility in *Rhododendron ellipticum*, *R. championae* and *R. amamiense*: A post-zygotic mechanism. Incompatibility Newsletter. 1984;16:10–1.

[90] Li S, Sun W, Ma Y. Current conservation status and reproductive biology of the giant tree *Rhododendron* in China. Nordic Journal of Botany. 2018;36(1):1–9. doi: 10.1111/njb.01999

